# Effects of Different Extraction Methods and Conditions on the Phenolic Composition of Mate Tea Extracts

**DOI:** 10.3390/molecules17032518

**Published:** 2012-03-02

**Authors:** Nevena Grujic, Zika Lepojevic, Branislava Srdjenovic, Jelena Vladic, Jan Sudji

**Affiliations:** 1 Department of Pharmacy, Medical Faculty, University of Novi Sad, Serbia; Email: srdjbr@yahoo.com (B.S.); vladicjelena@gmail.com (J.V.); 2 Department of Pharmaceutical Engineering, Faculty of Technology, University of Novi Sad, Serbia; Email: lepojevic@tf.uns.ac.rs; 3 The Institute for the Health Protection of Workers Novi Sad, Serbia; Email: sudy@neobee.net

**Keywords:** mate, chlorogenic acid, HPLC analysis, DPPH, total phenolic content, total flavonoid content

## Abstract

A simple and rapid HPLC method for determination of chlorogenic acid (5-O-caffeoylquinic acid) in mate tea extracts was developed and validated. The chromatography used isocratic elution with a mobile phase of aqueous 1.5% acetic acid-methanol (85:15, v/v). The flow rate was 0.8 mL/min and detection by UV at 325 nm. The method showed good selectivity, accuracy, repeatability and robustness, with detection limit of 0.26 mg/L and recovery of 97.76%. The developed method was applied for the determination of chlorogenic acid in mate tea extracts obtained by ethanol extraction and liquid carbon dioxide extraction with ethanol as co-solvent. Different ethanol concentrations were used (40, 50 and 60%, v/v) and liquid CO_2_ extraction was performed at different pressures (50 and 100 bar) and constant temperature (27 ± 1 °C). Significant influence of extraction methods, conditions and solvent polarity on chlorogenic acid content, antioxidant activity and total phenolic and flavonoid content of mate tea extracts was established. The most efficient extraction solvent was liquid CO_2_ with aqueous ethanol (40%) as co-solvent using an extraction pressure of 100 bar.

## 1. Introduction

*Ilex paraguariensis* St. Hil. (Aquifoliaceae) is a native plant of South America used for the preparation of tea-like beverage known as mate. During the recent years interest in mate is growing in many countries, mostly due to its antioxidant, stimulating, anti-obesity and anti-rheumatic pharmacological effects. A number of the phytochemical components have been identified in mate: phenols and phenol acids, flavonoids, triterpenoid saponins, minerals and purine alkaloids [1]. Aqueous and ethanol extracts of green and roasted yerba mate have shown excellent DPPH free radical scavenging activity. Significant content of chlorogenic, 3,5-dicaffeoylquinic, 4,5-dicaffeoylquinic, and 3,4-dicaffeoylquinic acids can be found in mate. This high polyphenolic content could be responsible for its antioxidant activity [2,3]. Chlorogenic acids (CGAs) are believed to be the main substances responsible for anti-glycation effect of *Ilex paraguariensis* [4]. Recent studies showed a high correlation between consumption of polyphenol-rich food and beverages and the prevention of many human diseases [5,6]. High-performance liquid chromatography (HPLC) with UV detection has been successfully used analytical method for determination of chlorogenic acid in herbal extracts. In most cases this methods involved gradient elution [7,8].

Liquid carbon dioxide is found to be a good extraction solvent in food industry due to its satisfactory physicochemical properties, low toxicity and price. Since CO_2_ is non-polar it is not suitable solvent for polar polyphenols. Addition of solvents like ethanol, methanol or acetone increases the solvating power of CO_2_ and the extraction yield of phenolic compounds. Ethanol is a permitted co-solvent in food industry [9]. In recent studies, total phenolic content was mainly determined in organic solvent and water herbal extracts [10,11] and little is known about polyphenolic content of ethanol+liquid CO_2_ herbal extracts.

Accordingly, the primary goal of this study was to develop a simple, rapid and reproducible HPLC method for chlorogenic acid determination in herbal extracts. A secondary goal was the extraction of mate (*Ilex paraguariensis*) tea leaves (solvent extraction and liquid CO_2_ extraction with co-solvent), identification and quantitation of chlorogenic acid and determination of antioxidant activity, total phenolic and total flavonoid content in obtained extracts.

## 2. Results and Discussion

### 2.1. Validation of the HPLC Method

**Figure 1:**
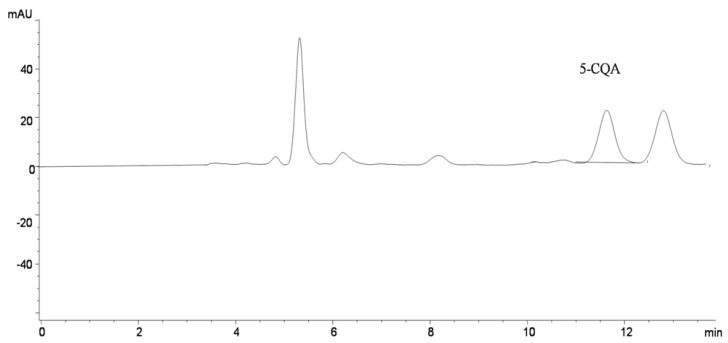
Representative HPLC-DAD chromatogram of mate tea extract obtained by liquid CO_2_ extraction with 40% ethanol using pressure of 100 bar.

The procedures used for validation of High-performance Liquid Chromatography (HPLC) methods for determination of 5-O-caffeoylquinic acid (5-CQA) in mate tea extracts are described in USP 24 [12] and in the International Conference of Harmonization Guidelines [13,14] or other sources [15]. Addition of acetic acid in a mobile phase composed of methanol and water is found to be good for sharpening peak shapes and improving analytical sensitivity and resolution for the HPLC analysis of phenolic compounds [8]. Selectivity of the method and linearity were evaluated. Limit of detection (LOD-signal to noise [S/N] ratio 3:1) and limit of quantitation (LOQ-S/N ration 10:1) were calculated and the results are presented in Table 1. As shown in Figure 1, there was no interference in the HPLC results by the other ingredients in tested sample, which indicate that developed method had good selectivity. The linearity relationship between concentrations and peak areas was excellent, with determination coefficient (r) of 0.999. Methods accuracy was determined by comparing measured and known values, analyzing working standard solutions of known concentrations. The mean recovery was 97.76% (n = 6 for each concentration) and it showed that presented method had good accuracy.

**Table 1:** Statistical parameters of 5-CQA calibration curve with LOD and LOQ.

5-CQA	
Linear range (mg/L)	10-100
Slope	251.8
Intercept	0.994
Determination coefficient (r)	0.999
Correlation coefficient (r^2^)	0.999
LOD (mg/L)	0.26
LOQ (mg/L)	0.87

A repeatability test was performed in order to estimate intra-day variation in the peak areas and retention times. Highest value for relative standard deviation (RSD) was 0.61% (n = 6) proving that repeatability of the method is satisfactory. An intermediate precision (inter-day repeatability) was determined over three-days analyzing working solutions under the same chromatographic conditions. Solutions were preserved on 4 °C and recovery ranged 100.25–96.85% over three days. The RSD values for migration time ranged 0.21–0.61% and for peak area ranged 0.78–1.35% showed acceptable intermediate precision of the analytical method. Robustness of the method was evaluated by analyzing the effects of slightly changed parameters of used HPLC conditions. Effects of different column temperature (±1 °C), flow rate (±0.05 mL/min) and detection wavelength (±3 nm) were observed. No significant variations were noticed for tested ranges which proved the good robustness of the analyzed HPLC method. 

### 2.2. Determination of Chlorogenic Acid in Mate Extracts

The compound peak obtained at 11.57 min (Figure 1) had identical retention time and UV spectra as a standard of chlorogenic acid. This was confirmed by adding a standard of chlorogenic acid to the analyzed sample. Considering the retention times and elution profiles in C18 columns reported for caffeic acid derivatives in mate [10,16], the compound corresponded to 5-*O*-caffeoylquinic acid (5-CQA). The results of 5-CQA in mate extracts obtained by extraction with aqueous ethanol (40–60%) and liquid carbon dioxide (CO_2_) extraction with aqueous ethanol (40–60%) as co-solvent are presented in Table 2. 5-CQA content approximately ranged from 0.21–1.66% of a dry extract. Obtained results are similar for ethanol extraction and higher for liquid CO_2_ extraction with ethanol than one obtained for 5-CQA content in mate infusions studied in recent publications [16,17]. This could be explained by higher selectivity of liquid CO_2_ with ethanol as co-solvent for chlorogenic acid. Also, it is well known that changes in solvent polarity used for the extraction alters its ability to dissolve a selected group of antioxidant compounds [18].

**Table 2:** 5-CQA content of mate (*Ilex paraguariensis*) leaves extracts.

Ethanol concentration(%, v/v)	5-CQA (g/100 g d.m.)		
	Extraction with ethanol	Liquid CO_2_ with ethanol extraction	
		50 bar	100 bar
40	0.25 ± 0.01 ^a^	0.36 ± 0.05 ^c^	1.66 ± 0.01 ^e^
50	0.22 ± 0.03 ^a^	0.31 ± 0.03 ^b^	1.61 ± 0.03 ^e^
60	0.21 ± 0.04 ^a^	0.26 ± 0.01 ^b^	1.52 ± 0.02 ^d^

Data are expressed as means ± standard deviations of triplicate measurements and expressed on a dry matter basis. Statistically significant differences are noted by different superscript letters (p < 0.05).

Alcoholic solvents (methanol and ethanol) have been commonly used to extract polyphenols from natural sources. Mixtures of alcohols and water have revealed to be more efficient in extracting phenolic compounds than compared to mono-component solvent. Addition of small amounts of water to organic solvents creates a more polar medium which facilitates phenolic extraction [19]. As shown in Table 2, for ethanol extraction, best results are obtained by using more polar solvent (aqueous ethanol, 40%). It is clear that using liquid CO_2_ with ethanol as co-solvent increases 5-CQA content in obtained extracts. Chlorogenic acid content increases as the function of liquid CO_2_ extraction pressure. 5-CQA amount was up to 4.61 times higher at 100 bar pressure then obtained at the pressure of 50 bar. It can be concluded that selectivity of liquid CO_2_ for chlorogenic acid increases with the pressure of extraction.

### 2.3. Antioxidant Activity, Total Phenolic and Flavonoid Content

Results of free radical scavenging activity, total phenolic and flavonoid content are presented in Table 3. Obtained mate tea extracts were tested for antioxidant activity using DPPH test. EC_50_ values, given in Table 3, indicate that all mate leaves extracts have certain antiradical activity. 

**Table 3:** Free radical scavenging activity (EC_50_), total phenolic content (TPC) and total flavonoid content (TFC) of mate (*Ilex paraguariensis*) leaves extracts.

Ethanol Concentration (%, v/v)	Extraction with Ethanol	Liquid CO_2_ with Ethanol Extraction	
		50 bar	100 bar
	EC_50_ (mg/mL)		
40	0.0157 ± 0.004 ^e^	0.0094 ± 0.000 ^b^	0.0034 ± 0.000 ^a^
50	0.0185 ± 0.001 ^f^	0.0113 ± 0.002 ^c^	0.0112 ± 0.001 ^c^
60	0.0385 ± 0.003 ^h^	0.0222 ± 0.001 ^g^	0.0132 ± 0.005 ^d^
	TPC (mg CAE/g d.m.)		
40	41.15 ± 0.18 ^c^	67.71 ± 0.25 ^f^	123.22 ± 0.85 ^h^
50	33.28 ± 0.66 ^b^	66.12 ± 0.33 ^f^	106.11 ± 0.73 ^g^
60	29.46 ± 0.23 ^a^	46.24 ± 0.78 ^d^	61.35 ± 0.55 ^e^
	TFC (mg CA/g d.m.)		
40	70.63 ± 0.13 ^c^	100.44 ± 0.21 ^f^	115.03 ± 0.28 ^g^
50	50.09 ± 0.21 ^b^	91.42 ± 0.14 ^e^	99.99 ± 0.33 ^f^
60	47.71 ± 0.09 ^a^	88.81 ± 0.11 ^d^	89.14 ± 0.25 ^d^

Data are expressed as the averages ± standard deviations of triplicate measurements and expressed on a dry matter basis. Different letters for EC_50_, TPC and TFC indicate significant difference on a level 0.05.

Results showed that the extraction method and conditions of extraction had significant effects on DPPH radical scavenging capacity of mate extracts. Extracts obtained using more polar extraction solvents are considerably more effective radical scavengers then those using less polar solvents [20]. In our research better results are obtained for liquid CO_2_ extraction with ethanol as co-solvent then using only ethanol. This is in accordance with previously published data [9]. When higher pressure in liquid CO_2_ extraction was used, antioxidant activity increased. This could be explained by the increase of carbon dioxide selectivity for antioxidant compounds with higher pressure [9]. 

Results of TPC for different mate extracts are presented in Table 3 and ranged from 3–12% of a dry extract. For the extraction with different concentrations of ethanol, significantly higher value was obtained for 40% ethanol, even 1.4 times value obtained for 60% ethanol. Results in Table 3 indicate that liquid CO_2_ extraction with ethanol as a co-solvent is more effective for polyphenols extraction then ethanol alone because of its increased polarity. Using ethanol with lower concentration as co-solvent significantly increases total phenolic content in mate extracts, so the best results are achieved for 40% ethanol. These results clearly showed that higher content of phenol compounds was obtained with an increase in solvent polarity used for the extraction. As the pressure increases, extraction of phenols from mate extracts is more efficient. Result for phenol compounds extracted on the pressure of 100 bar gives up to 1.82 times value of using the pressure of 50 bar. Presented results of this research were higher than one obtained in mate extracts when solvent was boiled water, 100% acetone, 100% DMF, 100% ethanol and 100% methanol [20]. This could be due to usage of less polar or non-selective solvents. Similar effect of extraction method and solvent polarity was noticed for total flavonoid content (TFC) of mate tea extracts. TFC increases in obtained extracts with increased polarity of extraction solvent and as the function of pressure in liquid CO_2_ extraction. In this research, best results for antioxidant activity, TPC and TFC were found in mate extracts obtained by liquid CO_2_ extraction with aqueous ethanol (40%) as co-sovent on a pressure of 100 bar. Our results confirmed previously published data finding aqueous ethanol (40%) to be the most efficient solvent for polyphenols extraction from green and white tea compared to other ethanol concentrations [21].

**Figure 2:**
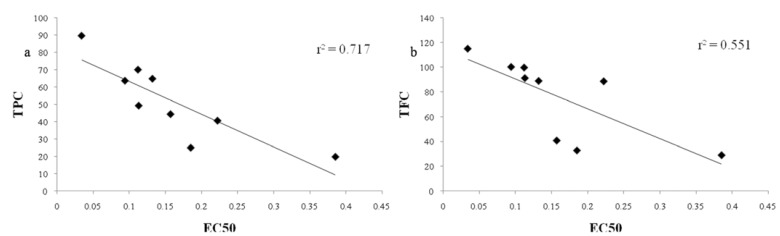
(**a**) Correlation of total phenolic content (TPC) and EC_50_ values of mate leaves extracts; (**b**) Correlation of total flavonoid content (TFC) and EC_50_ values of mate leaves extracts.

As shown in Figure 2a, very good correlation is established between antioxidant activity and total phenolic content with correlation coefficient of r = −0.85.

These results agree with the research finding correlation between antioxidant activity and phenolic content to be very good for black and mate tea [22]. Obtained results suggested that the phenolic compounds contributed significantly to the antioxidant capacity of *Ilex paraguariensis *extracts. The total phenolic content and antioxidant activity of mate were highly negatively correlated. Negative correlation implies that higher TPC leads to lower EC_50_ values and higher antioxidant potential.

There was less significant correlation between antioxidant activity and total flavonoid content (r = −0.74) (Figure 2b). These results are in agreement with other literature for green and black tea or other herbs [23,24]. It is known that only flavonoids with certain structures and hydroxyl position in the molecule can act as proton donors and this could be the reason for the weaker correlation between antioxidant activity and TFC of mate extracts [25,26].

## 3. Experimental

### 3.1. Chemicals

Carbon dioxide (99.99% purity) was purchased from Messer Tehnogas (Novi Sad, Serbia). The standard of chlorogenic acid (5-CQA, ≥95%), methanol, 1,1-Diphenyl-2-picrylhydrazyl-hydrate (DPPH), aluminium chloride, sodium hydroxide and sodium nitrite were obtained from Sigma Aldrich (St. Louis, MO, USA). Glacial acetic acid and ethanol (95–96%) were purchased from Zorka Pharma a.d. (Sabac, Serbia). Folin-Ciocalteu's reagent and (+)-catechin were obtained from Fluka BioChemika (Buchs, Switzerland) and anhydrous sodium carbonate from Sinex laboratory (Belgrade, Serbia). Ultra pure water was used for the preparation of all solutions. All solvents and reagents were of an analytical grade unless indicated otherwise.

### 3.2. Plant Material Extraction and Preparation

Mate *(Ilex paraguariensis)* tea leaves were obtained from Sinex company (Nis, Serbia). Before the extraction, leaves were ground in a mill and particle size diameter was determined by sieving (*d* = 0.5 mm). Moisture content of the samples was determined by drying at 80 °C for 24 h and it was found to be 10 ± 0.96%. Solvent extraction of mate tea leaves (10 g) was performed with 100 mL of ethanol/water solution on temperature 27 ± 1 °C for three hours. Different ethanol concentrations were used (40, 50 and 60%). Obtained extracts were filtered (Whatmen No.1) and 10 mL were used for the analysis. Solvent was evaporated prior the analysis. Liquid carbon dioxide extraction was carried out on laboratory-scale High Pressure Extraction Plant (HPEP, NOVA–Swiss, Effretikon, Switzerland). 10 g of a minced mate leaves were extracted with 100 mL of aqueous ethanol (40, 50 and 60%) as co-solvent in an apparatus extractor. CO_2_ under pressure was introduced in extractor with flow rate of 1.9 g/min. Extraction was performed on different pressures (50 and 100 bar) and on constant temperature (27 ± 1°C) for three hours. Ten mL of extracts were used for the analysis. All extracts were stored at +4 °C until analysis which was performed within one or two days.

### 3.3. HPLC Operating Conditions

The analytical determination of chlorogenic acid was carried out by HPLC using two-solvent isocratic elution. An Agilent HP 1100 HPLC-diode array detection (DAD) system equipped with an autosampler (Agilent, Waldbronn, Germany) was used. The analytical column was the Zorbax CB-C18 (4.6 × 150 mm, i.d., 5 µm particle size). Mobile phase was aqueous 1.5% acetic acid-methanol (85:15) with a flow rate of 0.8 mL/min. The HPLC mobile phase was prepared fresh daily and filtered through a 0.45 µm nylon filter. Run time was 15 min, column temperature 25 °C and analytes were detected at 325 nm.

### 3.4. Preparation of Stock and Working Standard Solutions for HPLC Analysis

**Figure 3:**
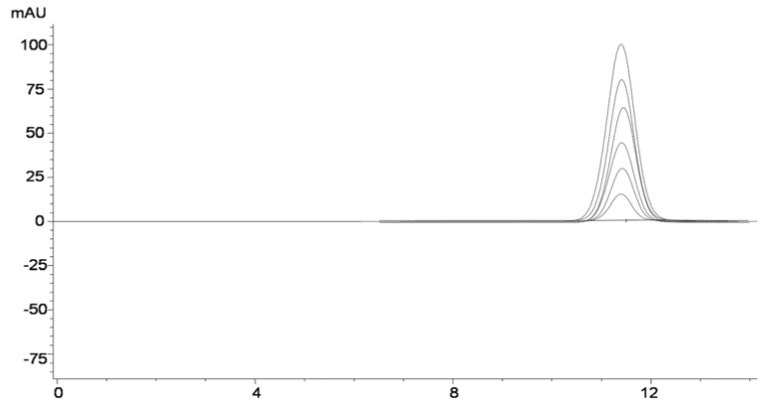
HPLC-DAD chromatogram of working standard solutions of 5-CQA (0.01–0.1 mg/mL) under optimized chromatographic conditions.

The standard stock solution of chlorogenic acid was prepared by weighing 10 mg of the standard substance and dissolving in 10 mL of methanol. The solution was stable approximately three days under refrigeration (4 °C). Working solutions were prepared by diluting 0.1, 0.3, 0.6, 0.8 and 1 mL of the stock solution to 10 mL with methanol to obtain different concentrations of chlorogenic acid (0.01–0.1 mg/mL). 10 µL of working solutions were injected into the HPLC system and the peak area responses were obtained (Figure 3). A method of the external standard calibration was used. Linear standard curve for chlorogenic acid was obtained by plotting concentration versus area. For determination of chlorogenic acid, extract was dissolved in 5 mL of methanol, 10 times diluted with the same solvent and filtered through a 0.45 µm nylon filter prior HPLC analysis. 10–20 µL of each sample were injected into HPLC system. All measurements were performed in triplicate. Chlorogenic acid content of the resulting extracts from ethanol extraction and liquid CO_2_ with ethanol extraction were compared.

### 3.5. Antioxidant Activity, Total Phenolic Content (TPC) and Total Flavonoid Content (TFC) Determination

Spectrophotometric measurements were performed using an Agilent 8453 UV-Visible Spectroscopy System (Germany). For sample preparation a Vortex-2 Genie (model: G-560 E, Scientific Industries, Inc. Bohemia, NY, USA) vortex mixer, was used. Antioxidant activity of extracts was determined using the DPPH method [27]. Herbal extracts were dissolved in methanol. Different volumes (10–50 μL) of herbal extracts were mixed in test tubes with 1,000 μL of DPPH solution (0.1 mM in methanol) and made up to final volume of 4 mL with methanol. Control sample was prepared containing the same volume without test compounds. Methanol was used as a blank. Absorbances were measured at 515 nm. The experiment was performed in triplicate and the average absorption was noted for each concentration. Radical Scavenger Capacity (%RSC) was calculated using the following formula:

%RSC = 100 × (A_control_ − A_sample_) / A_control_

The EC_50_ value, defined as the concentration of the test sample leading to 50% reduction of the free radical concentration, was calculated graphically and expressed as mg of the extract/mL of the final solution in measuring cell.

Total phenolic content (TPC) was determined using the Folin-Ciocalteu method [28]. A calibration curve of chlorogenic acid was prepared and the results were expressed as chlorogenic acid equivalents (mg CAE/g dry matter). Herbal extracts were dissolved in methanol. In this method herbal extract (0.1 mL), distilled water (7.9 mL), Folin-Ciocalteu’s reagent (0.5 mL) and saturated Na_2_CO_3_ solution (1.5 mL) were added into a test tube. A control sample was prepared at the same time using distilled water (8 mL), Folin-Ciocalteu’s reagent (0.5 mL) and saturated Na_2_CO_3_ solution (1.5 mL). Ingredients in test tubes were well mixed using the Vortex and left in a dark place for 2 hours. Absorbances were measured at 750 nm. All measurements were performed in triplicate.

Total flavonoid content (TFC) was evaluated according to a spectrophotometric assay with aluminium chloride [29]. An aliquot of mate extract (1 mL) was evaporated to dryness and dissolved in 20 mL of methanol. Methanol solution (1 mL) was added to a volumetric flask containing a solution of NaNO_2_ (0.3 mL, 0.5 g/L). After 5 min, a 1 g/L solution of AlCl_3_ (0.3 mL) was added and 6 min later, NaOH (2 mL, 1 mol/L) was added to the mixture. The total volume was made up to 10 mL with distilled water, the solution was mixed and the absorbance was measured at 510 nm against methanol blank. Catechin was used as the standard for the construction of a calibration curve and the concentrations are expressed as catechin equivalents (mg CE/g dry matter).

### 3.6. Statistical Analysis

The statistical analyses were performed by MS Excel^®^ 2007 software. Comparison of mean values of measured parameters was performed by a one-way ANOVA (SPSS, version 17) using Duncan’s multiple range test, for the level of significance p < 0.05.

## 4. Conclusions

It can be concluded that a simple and rapid HPLC method for determination of chlorogenic acid (5-CQA) was developed and validated. Obtained results for selectivity, accuracy, repeatability and robustness suggested that used method can be applied for identification and quantitation of chlorogenic acid in herbal extracts. Analysis of different mate tea extracts showed a great influence of the extraction methods and conditions and polarity of used extraction solvent on chlorogenic acid content, antioxidant activity and total phenolic and flavonoid content. The most efficient extraction solvent was liquid CO_2_ with aqueous ethanol (40%) using an extraction pressure of 100 bar. A high negative correlation was determined between radical scavenging activity and total phenolic content and less significant correlation between radical scavenging activity and total phenolic content of mate tea extracts was established.
